# Systemic Manifestation of Miliary Tuberculosis in Patient With Advanced Diabetic Retinopathy Presenting With Electrolyte Imbalance, Seizures, and Adrenal Insufficiency

**DOI:** 10.7759/cureus.21047

**Published:** 2022-01-09

**Authors:** Zahid Khan, Davina Jugnarain, Bashir Mahamud, Animesh Gupta, Shirin Patel, Gideon Mlawa

**Affiliations:** 1 Cardiology, Royal Free Hospital, London, GBR; 2 Medicine, East London NHS Foundation Trust, London, GBR; 3 Internal Medicine, Barking, Havering and Redbridge University Hospitals NHS Trust, London, GBR; 4 Acute Internal Medicine, Barking, Havering and Redbridge University Hospitals NHS Trust, London, GBR; 5 Internal Medicine/Diabetes and Endocrinology, Barking, Havering and Redbridge University Hospitals NHS Trust, London, GBR

**Keywords:** convulsive seizures, addison's disease, addisonian crisis, peripheral sensory neuropathy, diabetic retinopathy, diabetic nephropathy (dn), cerebellar tuberculoma, miliary tuberulosis

## Abstract

Extra-pulmonary manifestations of tuberculosis can have diverse presentations depending on the affected organs. In this case report, we describe a case of a 50-year-old man of South Asian origin who presented with acute adrenal crisis on a background of undiagnosed miliary tuberculosis. Imaging after repeated episodes of adrenal crisis and seizures revealed bilaterally enlarged adrenals and cerebral tuberculomas, suggesting adrenal and central nervous system involvement. CT chest, abdomen and pelvis showed apical lung nodules and tree-in-bud appearance suggestive of tuberculosis. Due to high endogenous levels of adrenocorticotropic hormone and a flat response after a short synacthen test, a diagnosis of primary adrenal insufficiency secondary to tuberculosis infection was made. He remains well on anti-tuberculous chemotherapy, corticosteroids, and anti-epileptic medication. This case report exemplifies the unusual but life-threatening presentations of extra-pulmonary tuberculosis that may become increasingly common with immunosuppression because of the human immunodeficiency virus global epidemic and immunosuppressant therapies; therefore, a low index of suspicion is needed in these cases.

## Introduction

Tuberculosis (TB) is one of the "big three" infectious diseases, caused by species belonging to the *Mycobacterium tuberculosis* complex (MTC) [1]. Primary and post-primary pulmonary TB are symptomatic where individuals classically present with a chronic productive cough, hemoptysis, and systemic symptoms of appetite loss, weight loss, fever, and night sweats [2]. Less commonly, extra-pulmonary TB (EPTB) can occur in 10-42% of patients, with risk factors such as immunosuppression and being of South-East Asian or African descent [3]. TB can involve other organs, leading to a diverse range of presentations, which can make it challenging to get the right diagnosis. EPTB is more common upon reactivation of latent primary TB. Impairment of host immunity is associated with extra-pulmonary spread, as this impairs containment of MTC enabling dissemination to other organ systems [4]. The most common sites involved in EPTB are lymph nodes (19% of cases), pleura (7% of cases), gastrointestinal tract (4% of cases), bone (6% of cases), central nervous system (CNS) (3% of cases), and miliary TB (3% of cases) [5]. 

## Case presentation

A 59-year old man of South Asian origin presented to the emergency department in January 2019, complaining of lethargy, vomiting, dysphagia, loss of appetite, progressive skin hyper-pigmentation, and 25 kg unintentional weight loss in the last eight months. His past medical history includes type 1 diabetes mellitus with retinopathy and nephropathy, hypertension, hypercholesterolemia, and ischemic heart disease with a previous coronary artery bypass graft in 2007. On examination, he had hyperpigmentation of the buccal mucosa and he was tachycardic, tachypnoeic, and hypotensive. For initial correction of hyponatremia, hyperkalemia, and metabolic acidosis, fluid resuscitation was commenced, and due to suspected adrenal insufficiency, he was prescribed 40 mg prednisolone once daily indefinitely (Table 1).

**Table 1:** Biochemical and hematological laboratory result for patient

Blood test	July 26, 2019	August 8, 2019	August 29, 2019	October 8, 2019	January 27, 2020	Normal range
Hemoglobin	112	109	105	118	120	133-173 g/L
White cell count	5.5	5.1	5.4	5.3	6.6	3.8-11 x 10^9^/L
Sodium	124	127	130	134	139	133-146 mmol/L
Potassium	4.6	5.3	5.3	5.3	5.0	3.5-5.3 mmol/L
Urea	14.1	14.2	14.1	15.6	7.2	2.5-7.8 mmol/L
Creatinine	182	206	218	174	150	62-106 umol/L
Calcium	2.73	2.74	2.63	2.62	2.47	2.2-2.6 mmol/L

**Figure 1:**
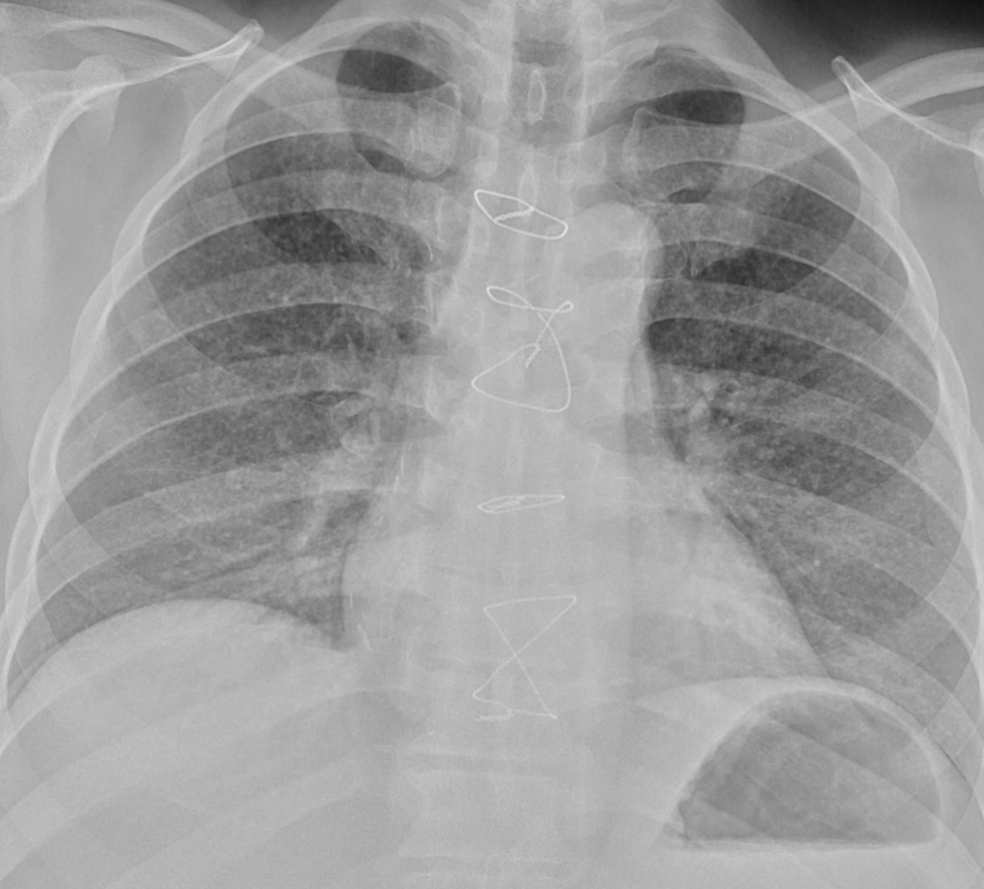
Chest radiograph showing miliary TB features TB: tuberculosis

**Figure 2:**
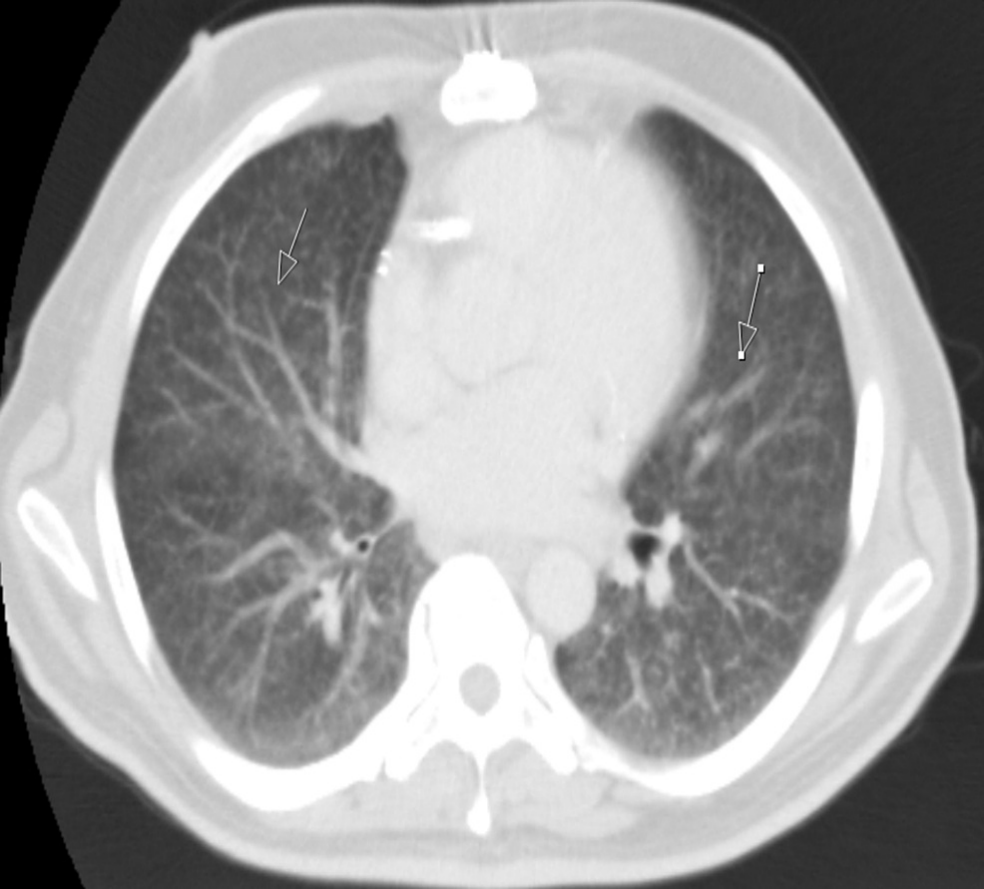
CT CAP showing tree-in-bud appearance in both lungs CAP: chest, abdomen and pelvis

His chest radiograph demonstrated miliary shadowing bilaterally in his lung fields (Figure 1), and computed tomography (CT) imaging revealed apical lung nodules, tree-in-bud appearance, and lymphadenopathy (Figure 2). The patient was given an imaging diagnosis of miliary TB and treatment was initiated while awaiting sputum analysis. He was treated with rifampicin 600 mg once daily, isoniazid 300 mg once daily, pyrazinamide 1 g once in the morning, and moxifloxacin 400 mg once daily in place of ethambutol to reduce the risk of optic neuritis due to his background of diabetic retinopathy. For miliary TB, the treatment duration was extended to one year, with cessation of pyrazinamide after two months. She also received pyridoxine 20 mg once daily to minimize the risk of peripheral neuropathy.

**Figure 3:**
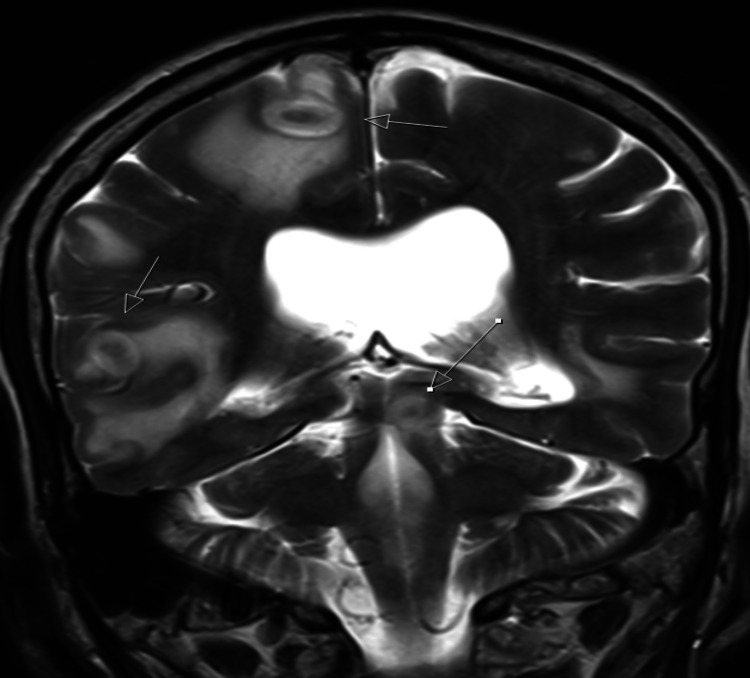
MRI brain showing tuberculomas

**Figure 4:**
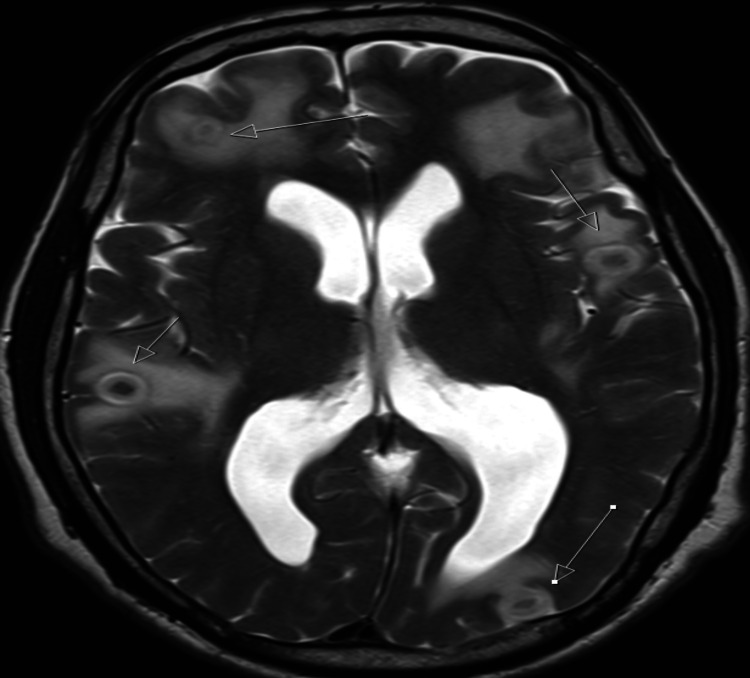
MRI showing multiple tuberculomas

By early February 2019, the patient had responded well and regained weight. At this point, we wanted to wean off his corticosteroid treatment to explore the effects of adrenocorticotropic hormone (ACTH) stimulation by short synacthen test to investigate adrenal insufficiency as a cause for his initial presentation. However later that month, the patient was admitted following a minute-long tonic-clonic seizure, with a post-ictal phase of one hour. He also experienced headaches and personality change, all indicating central nervous system (CNS) involvement. The patient underwent CT and magnetic resonance imaging (MRI) of the brain, revealing multiple enhancing lesions in the cerebrum, cerebellum, and brainstem, consistent with multiple tuberculomas (Figures 3, 4). As a result, dexamethasone 8 mg was initiated on a weaning regime to control tuberculoma development, and levetiracetam 500 mg twice daily was initiated for seizure control.

In March 2019, he represented to the hospital with vomiting, weakness, oliguria, and hypotension. The day prior the patient had stopped dexamethasone as instructed. Laboratory investigation revealed a normal anion gap metabolic acidosis, hyponatremia, hyperkalemia, and hyperuricemia, suggestive of hypovolaemia and renal tubular acidosis. In the intensive care unit, he was managed with hemofiltration and sodium bicarbonate to manage hyperkalemia and metabolic acidosis, respectively. CT and MRI of the abdomen revealed bilaterally enlarged adrenals. Random cortisol was 534 nmol/L, low for an acutely unwell patient, suggesting acute adrenal crisis potentially secondary to adrenal tuberculosis. The patient presented a further three times over the next five months after weaning corticosteroids, requiring double his normal dose of corticosteroids and IV fluids for improvement.

In July 2019, the patient was successfully weaned off hydrocortisone for one month. The patient reported general malaise, loss of appetite, and weight loss; however, this allowed a short synacthen test in July and August 2019. Both demonstrated a flat cortisol response of approximately 200 nmol/L (Table 2). Given a high serum ACTH of 1179 ng/L, this confirmed a primary adrenal insufficiency, and hydrocortisone was restarted. In this case, the seizures and adrenal insufficiency experienced by this patient are likely EPTB manifestations within the CNS and adrenals, respectively, resulting from the hematogenous spread of high-burden miliary TB.

**Table 2:** Short synacthen test result

Short synacthen test	Serum cortisol (nmol/L)		
	0 mins	30 mins	60 mins
July 2019	217	216	206
August 2019	199	196	182

## Discussion

In miliary TB, a common EPTB manifestation is within the central nervous system (CNS), manifesting as TB meningitis or encephalitis, which may be accompanied by cerebral space-occupying lesions (tuberculomas). Presentations, therefore, include altered consciousness, headaches, seizures, and focal neurological deficits depending tuberculoma site [4]. Davis et al. in 2019 described CNS involvement in TB as a two-step model, where hematogenous spread seeds MTC in meninges or cerebral parenchyma establishing caseous tuberculomas, which can develop into abscesses that rupture into the subarachnoid space causing tuberculous meningitis [6].

Adrenal TB can present insidiously with metabolic derangements and symptoms of adrenal insufficiency [4], such as lethargy, weakness, appetite loss, weight loss, nausea, vomiting, and progressive skin hyperpigmentation [7]. It classically manifests once over 90% of the adrenals have been damaged by tuberculosis, and therefore adrenocortical function may be normal initially, complicating detection of subclinical adrenal TB [8]. Interpretation of cortisol responses to synacthen testing should be done in the context of serum ACTH and albumin levels and, if a high index of suspicion, adrenal biopsies [7]. Another non-specific sign is the enlargement of adrenals which can be a marker of active adrenal TB or simply an infection-induced stress response [7]. Lack of restoration of adrenocortical function after TB chemotherapy can also be suggestive of adrenal TB, although adrenal insufficiency may be overt by this stage [9]. In fact, as a hepatic cytochrome P450 enzyme inducer, rifampicin accelerates corticosteroid metabolism, and has previously precipitated acute adrenal crisis [10]. Therefore high-dose hydrocortisone is advisable in patients with adrenal insufficiency with suspected direct TB involvement [7, 8].

This case report describes the progression of long-standing miliary tuberculosis to cerebral tuberculoma and adrenal tuberculosis. Immunosuppressive therapies and the HIV/AIDS pandemic are likely to increase the incidence of unusually presenting extra-pulmonary manifestations of tuberculosis. Therefore, it is important to acknowledge adrenal insufficiency and seizures as potential presentations of tuberculosis, particularly in the context of disseminated miliary tuberculosis, and to understand what can be done to detect them in their subclinical stages.

## Conclusions

In conclusion, this case report presents the complexity of managing a patient with multiple comorbidities and advanced diabetes with complications with active tuberculosis. It is important to be mindful of the fact that patients with tuberculomas are at risk of seizures and should be carefully managed. In addition, this case was complicated by the fact that the patient had an Addisonian crisis on admission and was also given steroids. She had advanced diabetic retinopathy and nephropathy which makes the management of tuberculosis more challenging.

It is important to manage these patients through a multidisciplinary team pathway due to the complexity of the situation and neurologists, nephrologists, diabetes and endocrine specialists, and respiratory specialists should be involved in the case of these patients. These patients are at risk of Addisonian crisis and steroid doses may need to be increased temporarily when they are unwell. Immunosuppressive therapies and the current coronavirus disease 2019 (COVID-19) pandemic are likely to increase the incidence of unusually presenting extra-pulmonary manifestations of tuberculosis such as adrenal insufficiency and seizures, and it is important to be aware of the management strategies in such cases.
